# Self-healing oxygen evolution catalysts

**DOI:** 10.1038/s41467-022-28723-9

**Published:** 2022-03-10

**Authors:** Agnes E. Thorarinsdottir, Samuel S. Veroneau, Daniel G. Nocera

**Affiliations:** Department of Chemistry and Chemical Biology, Harvard University, Cambridge, MA 02138 USA

**Keywords:** Energy, Electrocatalysis, Catalytic mechanisms

## Abstract

Electrochemical and photoelectrochemical water splitting offers a scalable approach to producing hydrogen from renewable sources for sustainable energy storage. Depending on the applications, oxygen evolution catalysts (OECs) may perform water splitting under a variety of conditions. However, low stability and/or activity present challenges to the design of OECs, prompting the design of self-healing OECs composed of earth-abundant first-row transition metal oxides. The concept of self-healing catalysis offers a new tool to be employed in the design of stable and functionally active OECs under operating conditions ranging from acidic to basic solutions and from a variety of water sources.

## Abstract (web summary)

Large scale sustainable energy storage by water splitting benefits from performing the oxygen evolution reaction under a variety of conditions. Here, the authors discuss self-healing catalysis as a new tool in the design of stable and functionally active catalysts in acidic to basic solutions, and a variety of water sources

## Introduction

Large-scale implementation of sustainable energy is needed to address the rising energy demands of our society while avoiding the detrimental impacts of fossil fuels. Whereas sustainable energy supplies (e.g., solar energy) are abundant, their implementation is bottlenecked by the challenge of storing this energy at the scale of societal demand^1–3^. One potentially scalable approach to energy storage is the production of hydrogen gas (H_2_) through renewably-driven electrochemical water splitting^1,2,4,5^. Hydrogen is a reliable energy carrier that can be used directly as a green fuel^2^, or be employed to furnish increasingly energy-dense products, including liquid fuels^6–9^ ammonia^10,11^. Water splitting (Eq. (1)) is an endergonic process that demands an external energy input greater than the thermodynamic minimum of 1.23 V to proceed:

1 $$2{{{{{{\rm{H}}}}}}}_{2}{{{{{\rm{O}}}}}}\to {{{{{{\rm{O}}}}}}}_{2}+2{{{{{{\rm{H}}}}}}}_{2}\;{E}^{{{{{{\rm{o}}}}}}}={{E}}_{{{{{{\rm{HER}}}}}}}^{{{{{{\rm{o}}}}}}}-{{E}}_{{{{{{\rm{OER}}}}}}}^{{{{{{\rm{o}}}}}}}=-1.23\,{{{{{\rm{V}}}}}}$$

2 $${{{{{{\rm{2H}}}}}}}_{2}{{{{{\rm{O}}}}}}\to {{{{{{\rm{O}}}}}}}_{2}+4{{{{{{\rm{H}}}}}}}^{+}+4{{{{{{\rm{e}}}}}}}^{-}\;{{E}}_{{{{{{\rm{OER}}}}}}}^{{{{{{\rm{o}}}}}}}=1.23{{{{{\rm{V}}}}}}-0.059{{{{{\rm{V}}}}}}\times {\rm {pH}}$$

3 $$4{{{{{{\rm{H}}}}}}}^{+}+4{{{{{{\rm{e}}}}}}}^{-}\to 2{{{{{{\rm{H}}}}}}}_{2}\;{{E}}_{{{{{{\rm{HER}}}}}}}^{{{{{{\rm{o}}}}}}}={{{{{\rm{0V}}}}}}-0.059\,{{{{{\rm{V}}}}}}\times {{{{{\rm{pH}}}}}}$$

Thus, renewable energy, whether it be from solar or wind, may be stored in the rearranged bonds of water as H_2_ and O_2_^1^. Of the two half-reactions of water splitting (Eqs. (2), (3)), the 4e^–^/4H^+^ oxygen evolution reaction (OER) is particularly demanding^12^. The most active and stable oxide catalysts for OER comprise critical metals (i.e., Ru, Ir)^13^. However, with regard to commercially relevant applications, they are not stable enough^14,15^ and they are costly owing to their scarcity. These challenges provide an imperative for the development of oxygen evolution catalysts (OECs) from earth-abundant elements that are both highly active and stable in various water sources under a range of operating conditions^16–18^.

Earth-abundant first-row transition metal oxides, however, have been relegated to operation in concentrated base^13,19^. The presence of base is required because metal oxides are themselves basic according to the Lux classification^20^ and will readily react with protons produced through OER (Eq. (2)), leading to damage (i.e., dissolution, corrosion, protonation). In concentrated base, hydroxide (OH^−^) is the strongest base and will neutralize these protons to protect the oxide; however, in less basic solutions, the concentration of OH^−^ is not sufficient making the primary base the oxide itself, leading to catalyst damage and inactivation. The ability to operate in nonbasic conditions has advantages of using natural water sources^21^, facilitating the interfacing of catalysts with materials such as Si^22–26^, which is unstable in corrosive basic conditions, reducing liability associated with technology advancement especially for distributed systems, and enabling the interfacing of water splitting catalysis to bio-organisms in hybrid inorganic–biological devices^8,9,27,28^. A unique strategy for operating earth-abundant first-row transition metal oxide OECs outside of strongly basic conditions is through the implementation of self-healing.

Within the catalysis community, multiple definitions of self-healing and self-repairing catalysts have been proposed^29,30^. The concept of self-healing has historically been used to describe any material with the ability to repair itself. Materials ranging from bio-inspired systems^31,32^ to synthetic organic polymers^33–35^, inorganic–organic hybrid materials, and metallic systems^36–38^ have all been considered self-healing. This can be through autonomous or stimuli-triggered processes that occur without or with external input (e.g., energy, pressure, chemical healing agents), respectively^34–38^. Many materials described as self-healing, including bio-inspired synthetic materials and metallic systems, however, induce repair after significant functional damage has already occurred, involve processes outside of normal operation, or rely upon the presence of chemical healing agents that are continually depleted^34–38^. We restrict the term “self-healing” herein to describe systems that continually regenerate themselves through a dynamic equilibrium during catalysis and under a given set of operating conditions. For example, self-healing notably differs from the repair mechanism of the Oxygen-Evolving Complex in Photosystem II, where the damaged reaction center is continuously replaced by a newly synthesized copy^39,40^. Furthermore, based on our criteria, catalyst regeneration mechanisms that rely on chemical oxidants (e.g., Ce^4+^)^41^, require activation by light to release oxygen and regenerate the precatalytic resting state^42^, or involve regeneration at conditions outside of that required for catalysis^43^ are considered self-repairing but not self-healing. Self-repairing metal oxide-based OECs have been comprehensively reviewed^44^.

**Fig. 1:**
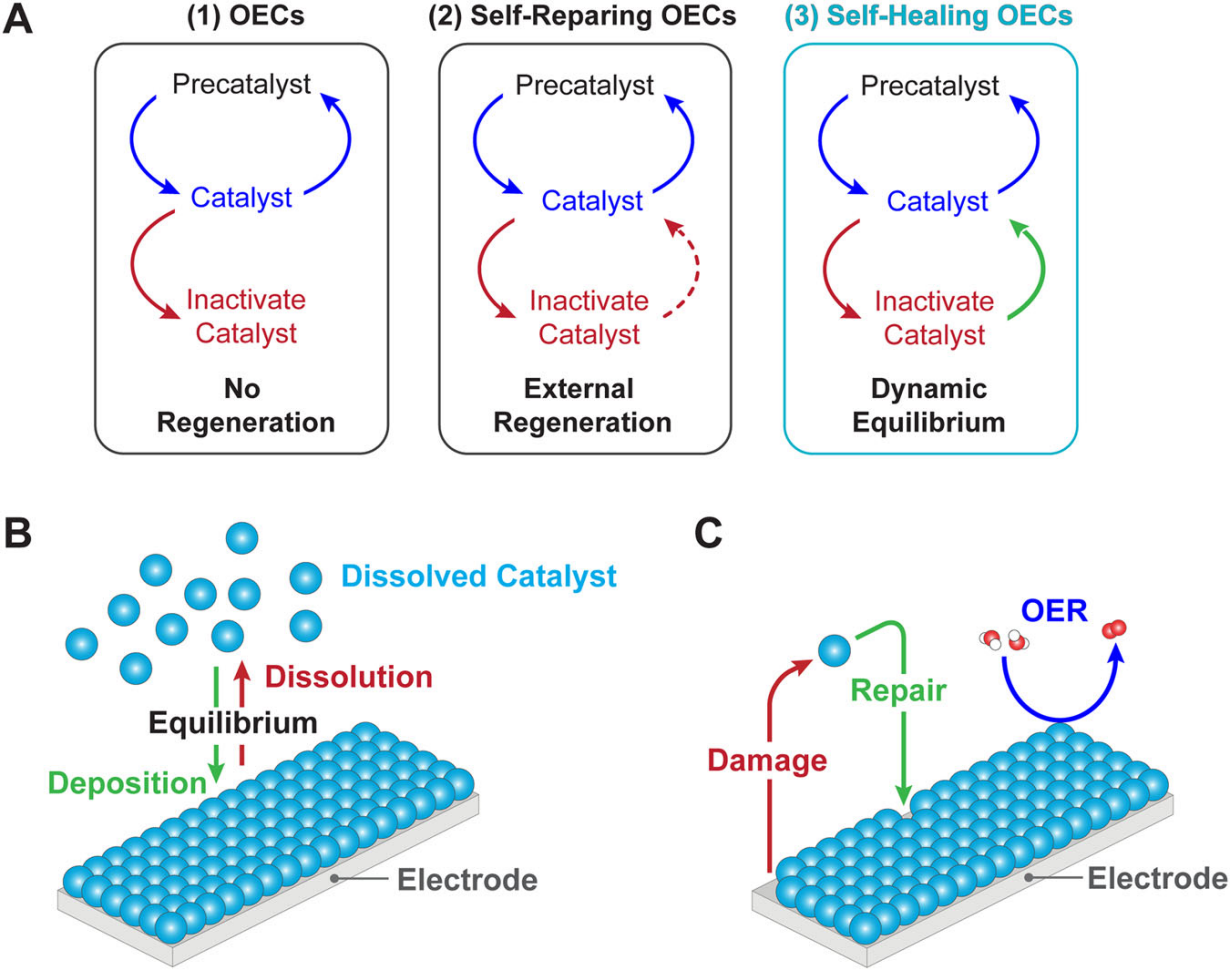
Schematic depiction of self-healing OECs. **A** Self-repairing OECs (center) are a specific type of OECs (left) that may operate for a prolonged time as they are regenerated once they become inactive, usually with the aid of an external input (e.g., energy, pressure, chemical healing agents). Self-healing OECs (right) are a specific type of OECs that continually regenerate themselves through an equilibrium process that occurs under the operating conditions of OER. **B** Graphical representation of the competing effects of catalyst deposition and dissolution that give rise to the equilibrium implicit for self-healing OECs. **C** Based on the equilibrium shown in (**B**), a damaged site is continuously repaired during OER for self-healing OECs, and as the rate of repair is greater than or equal to the rate of damage, no loss of catalytic species is observed. Blue spheres represent a catalytically competent metal capable of self-healing (e.g., Mn, Co, Ni, Cu).

Figure 1 schematically contrasts OECs that degrade with ones that self-repair and self-heal. Electrochemical and photoelectrochemical OER relies upon applying an external bias (potential) to the OEC to promote reactivity (Eq. (2)). Self-healing OECs are realized when this external bias is sufficient to drive regeneration such that the rate of repair is greater than or equal to the rate of damage. The required potential for this process is therefore less than or equal to the potential required to drive OER (Fig. 1C). This requirement is not fulfilled by self-repairing OECs, thus distinguishing self-healing from self-repairing catalysts. This is illustrated schematically in Fig. 1B where metal ions at equilibrium in solution can (re)deposit onto the still active and functional catalyst, assuming adequate mass transport from the solution to the electrode surface^30^. At equilibrium, it may be considered that there is no net degradation of catalyst, to reconcile a previous definition of self-healing^29^. We emphasize that self-healing as defined here *is not established universally by structure/composition but rather is defined operationally*. For oxide catalysts, structural and compositional damage occurs most commonly by protonation of surface oxo-species and the subsequent amorphization or dissolution of surface metal species; degradation mechanisms of OECs may also involve other structural rearrangement, mechanical, and poisoning effects^45^. The ability of the catalyst to regenerate itself will depend on the types and amounts of electrolytes and buffers, bulk pH values, applied currents and potentials, temperature, mass transport conditions, etc. Consequently, a homogeneous or heterogeneous OER catalyst of a given composition, crystallinity, or polymorphism may degrade under one set of conditions and not another. Thus, self-healing is determined by the set of conditions in which the catalyst operates.

We now survey the current state of OECs based on first-row transition metal oxides that exhibit self-healing behavior. Dimensional reduction of first-row metal oxides of Mn, Co, and Ni gives rise to metallate oxygen evolution catalysts (M-OECs) that exhibit high activity for OER^46–49^. We focus on the kinetics of OER and self-assembly, which form the foundation for the inherent self-healing properties of M-OECs. We highlight how the distinct kinetics of these processes determine the stability and activity of catalysts under different operating conditions, how the concept of self-healing is extended to multimetallic systems and discuss future approaches to developing increasingly active self-healing OECs.

## Self-healing Co-OECs

Cobalt-based M-OECs (Co-OECs) in the presence of phosphate (P_i_) and borate (B_i_) are exemplar self-healing OECs (Co-OECs generated in the presence of P_i_ or B_i_ are referred to hereafter as CoP_i_ and CoB_i_, respectively). These catalysts are generated as amorphous thin films on various conductive substrates (e.g., indium tin oxide, fluorine-doped tin oxide) during anodic electrodeposition from dilute aqueous Co^2+^ solutions in the presence of P_i_^50^, methylphosphonate (MeP_i_)^51^, or B_i_^21,52^ electrolytes in neutral to mildly basic conditions. These oxoanions facilitate the dimensional reduction of extended metal oxides by capping cluster growth to give rise to metallate active sites, which range from 10 to 60 metal centers, as deduced from X-ray pair distribution function (PDF) analysis of heterogeneous films^52–55^. The molecular nature of the M-OECs has allowed the mechanism of OER catalysis to be defined at a molecular and atomistic level. Isotopic measurements using differential electrochemical mass spectrometry^56^ together with electrokinetics^51^, spectroscopic^57^, and computational^58,59^ studies establish that O–O bond formation occurs by proton-coupled electron transfer (PCET) at cobaltate cluster edge sites.

**Fig. 2:**
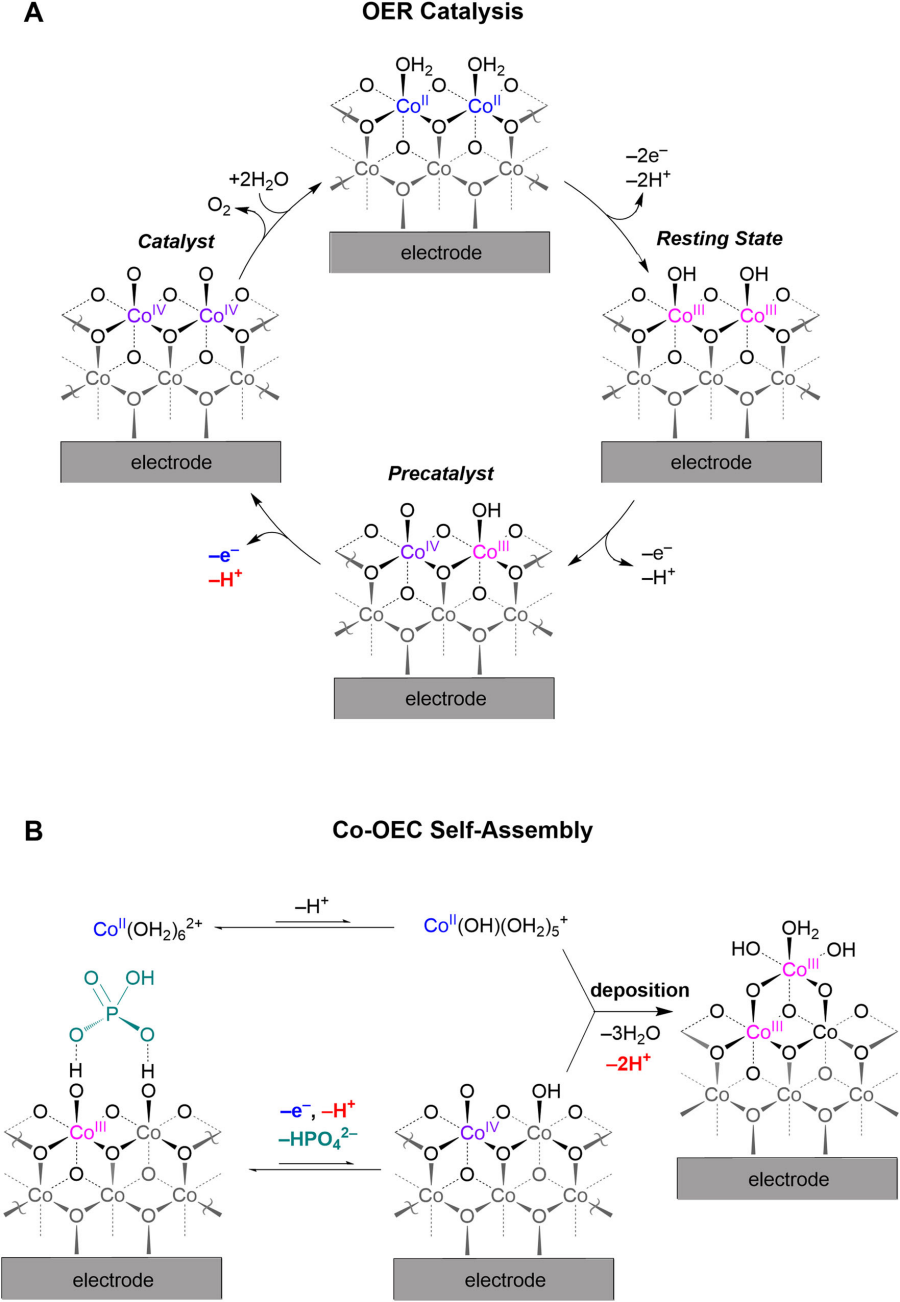
Co-OEC catalysis and self-assembly. **A** Proposed OER catalytic cycle (top cycle in Fig. 1A (3)) for Co-OECs (i.e., CoP_i_, CoMeP_i_, CoB_i_), as determined from spectroscopic analysis and electrochemical kinetics studies for CoP_i_. **B** Proposed mechanism for the generation of Co-OEC films (bottom cycle in Fig. 1A (3), and green arrows in Fig. 1B, C). The protons (H^+^) that appear in the electrochemical rate laws for OER and catalyst deposition/ regeneration are highlighted in red.

Figure 2A shows the mechanism for OER catalyzed by Co-OECs. The catalyst resides in a Co^III^Co^III^ resting state (vide infra), as highlighted in Fig. 2B, from which the Co^III^Co^IV^ precatalytic state is generated. Here, the terminal Co^IV^–oxo (i.e., Co^IV^ = O) is better formulated as a Co^III^–oxyl (i.e., Co^III^–O•) radical based on the electronic considerations embodied by the “oxo wall”^60^. Tafel analysis at pH 7 reveals a slope of 59 mV dec^−1^, indicating that the active Co^IV^Co^IV^ catalyst is generated by a 1e^–^/1H^+^ pre-equilibrium step followed by a turnover rate-limiting chemical step involving O–O bond formation and O_2_ release^61^. Accordingly, the rate of OER possesses an inverse first-order dependence of log(*j*_OER_) on proton activity (i.e., first-order dependence on pH), as summarized in the following electrochemical rate law obtained from electrokinetics studies:

4 $${j}_{{{{{{\rm{OER}}}}}}}={{k}_{0}}^{{{{{{\rm{OER}}}}}}}{({a}_{{{{{{\rm{H}}}}}}+})}^{-1}\exp ({\mathrm F}E/{\mathrm R}T)$$

where *j*_OER_ is the current density for OER and *k*_0_^OER^ is a potential-independent constant that is proportional to the exchange current density for OER.

Self-healing is established between the interplay of the potential required for OER and that required for self-assembly (i.e., deposition and regeneration) of the Co-OECs. Electrochemical kinetics have revealed the mechanism for catalyst deposition/regeneration to be as shown in Fig. 2B. At the deposition potential of 1.0 V vs. the normal hydrogen electrode (NHE) there is a minor Nernstian population of Co^4+^, as verified by EPR spectra of CoP_i_ films^62^, and consistent with the redox Co^3+^/Co^4+^ midpotential^63^. The Co^4+^ on the electrode oxidizes Co^2+^ in solution, which is in the form of Co(OH)(OH_2_)_5_^+^ at the 1.0 V deposition potential, by inner-sphere electron transfer upon dissociation of P_i_, giving rise to substitutionally inert Co^3+^ that adds to the exposed edge sites for metallate cluster growth. The pH dependence of CoP_i_ film deposition reveals an inverse third-order dependence on proton activity (i.e., third-order dependence of log(*j*_OER_) on pH), as well as an inverse first-order dependence on [P_i_], and first-order dependence on [Co^2+^], owing to the need to dissociate P_i_ for the inner-sphere electron transfer between Co^4+^ on the surface and Co(OH)(OH_2_)_5_^+^ in solution^64^. Thus, the overall rate law for deposition of CoP_i_ is^65^:

5 $${j}_{{{{{\rm{dep}}}}}}={k}_{0}^{{{{{\rm{dep}}}}}}[{\rm {C{o}}}^{2+}]{({a}_{H+})}^{-3}{[{{\rm {P}}}_{{\rm {i}}}]}^{-1}\exp ({\mathrm F}E/{\mathrm R}T)$$

where *j*_dep_ is the current density for catalyst deposition/regeneration and *k*_0_^dep^ is a potential-independent constant that is proportional to the exchange current density for the deposition/regeneration process.

 Self-healing is achieved because the potential needed to drive CoP_i_ film self-assembly (i.e., produce Co^4+^ for deposition) occurs at 0.2 V below the potential required for sustaining OER. Accordingly, potentials sufficient to sustain OER will oxidize any Co^2+^ in solution that may have leached from the film during operation and, thus, induce instantaneous redeposition. The transport of Co^4+^ in CoP_i_ films (i.e., the oxidizing hole equivalent) is predicted to be fast based on the Co^3+/4+^ self-exchange electron transfer rate constant of *k*_ET_(Co^3+/4+^) = 3 × 10^5^ M^–1^ s^–1^ as measured in cobalt cubane model complexes^66^. Because such hole hopping through the film is fast relative to Co^2+^ deposition^62,67^, very little Co^2+^ is produced in solution. Nonetheless, as confirmed by Co-57 radiolabeling of CoP_i_ films, any Co^2+^ released in solution is redeposited via the mechanism shown in Fig. 2B^68^. Consequently, no film dissolution is observed during OER when P_i_ is present at intermediate-to-high concentration (>~1 mM). Furthermore, this self-healing mechanism also applies for Co-OECs with MeP_i_ and B_i_ oxoanions, and similar self-healing Co-OECs are posited to form from the decomposition of molecular cobaloxime precursors in B_i_ buffer solutions (pH 9.2) upon application of high positive potentials^69^.

**Fig. 3:**
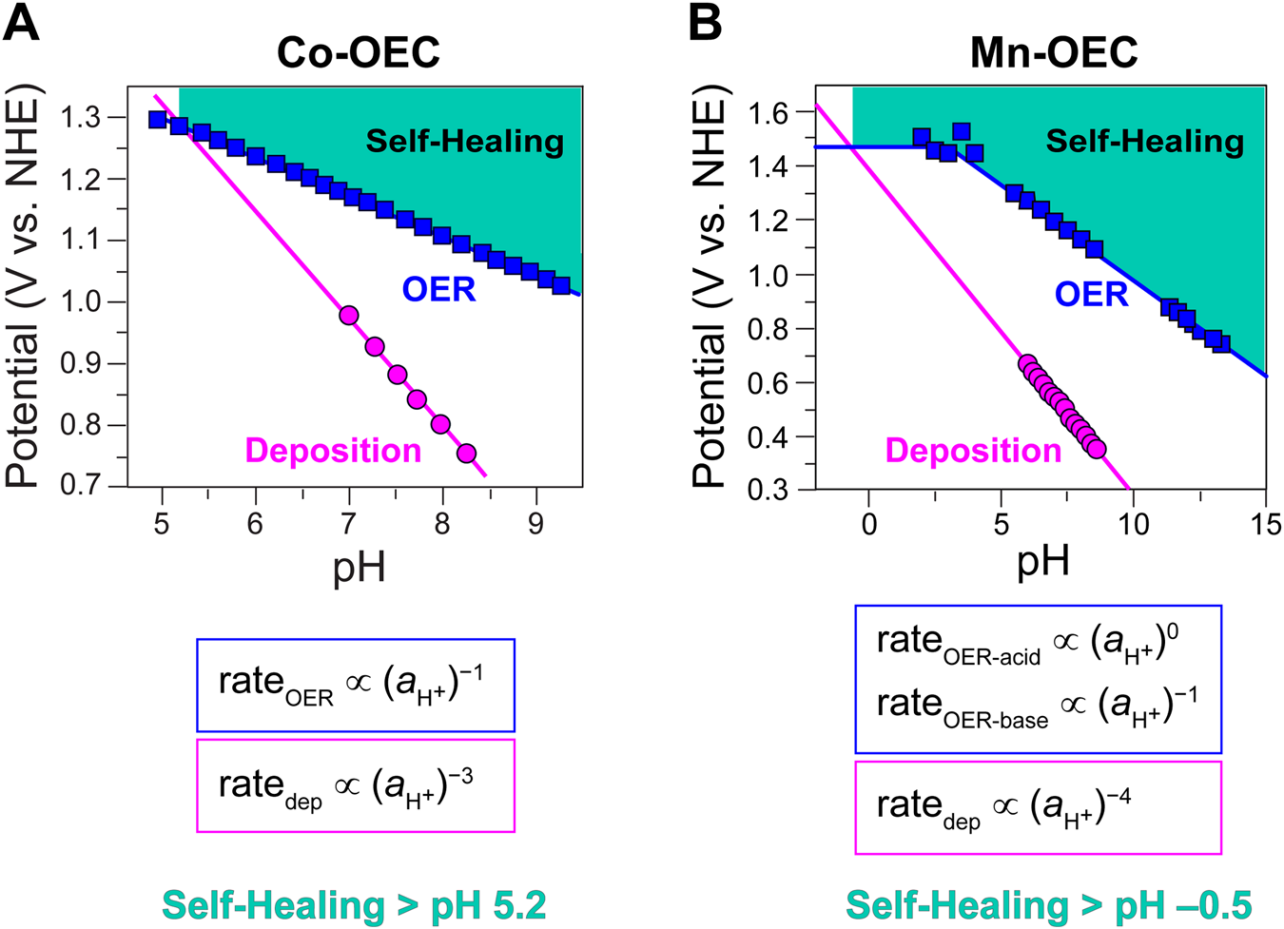
Pourbaix diagrams for Co- and Mn-OECs. Potential–pH diagrams for (**A**) Co-OECs and (**B**) Mn-OECs at fixed current densities of *j* = 30 and 1.3 μA cm^−2^ (based on geometric electrode surface area), respectively. The dependence of the rate of OER and the rate of catalyst deposition/regeneration on proton activity (*a*_H+_) are highlighted in blue and magenta, respectively, for each catalyst type. The different pH dependence for the two processes forms the foundation for the self-healing properties of Co-OECs and Mn-OECs above pH 5.2 and −0.5, respectively, in solutions devoid of component metals as indicated with the teal zones in the graphs. All potentials are referenced to the NHE scale.

The disparate pH profiles for OER and catalyst generation for Co-OECs give rise to a “Pourbaix” diagram for self-healing. Because of the steeper inverse third-order dependence on proton activity for deposition, the potential necessary for catalyst self-assembly rises much more rapidly with decreasing pH as compared to that for OER. As highlighted by the teal zone in Fig. 3A, the potentials necessary to sustain catalyst film formation are well below those required for OER. Thus, as long as Co-OECs are operated in the teal zone in Fig. 3A (pH > 5.2) the catalysts are indefinitely stable in aqueous solutions under the operational conditions that promote self-healing. This functional stability range for CoP_i_ is further supported by the observation of catalyst damage at pH 5 and below^47,65^. By introducing 0.1–1 mM Co^2+^ to the buffered electrolyte, however, Co-OECs may remain functionally stable down to pH of ~3 as the increased concentration of Co^2+^ drives a dynamic equilibrium, as shown in Fig. 2B, toward catalyst deposition and thus regeneration^70,71^. Below pH ~3, however, these low concentrations of Co^2+^ are insufficient to offset metal oxide dissolution and catalyst damage cannot be reversed. Notably, operational stability of Co-OECs at pH 1.6 can be achieved at higher Co^2+^ concentrations of 0.6 M in phosphate or sulfate electrolytes under application of potentials above 2.05 V vs. NHE^72^.

The CoP_i_ OER catalyst is exemplary of the concept of self-healing from an operational as opposed to a structural or compositional viewpoint. As emphasized for CoP_i_, self-healing is not achieved when the buffer concentration is too low^29,71^, as the rate of catalyst redeposition is smaller than the rate of dissolution. Hence under one set of conditions (e.g., low P_i_ concentration or a given current, potential, etc.) CoP_i_ is self-repairing, while under a different set of conditions that establishes Fig. 1C, CoP_i_ is self-healing. As a metric, a self-healing catalyst under operating conditions has a turnover number that is extremely large and in the limit approaches infinity.

## Self-healing Mn-OECs

**Fig. 4:**
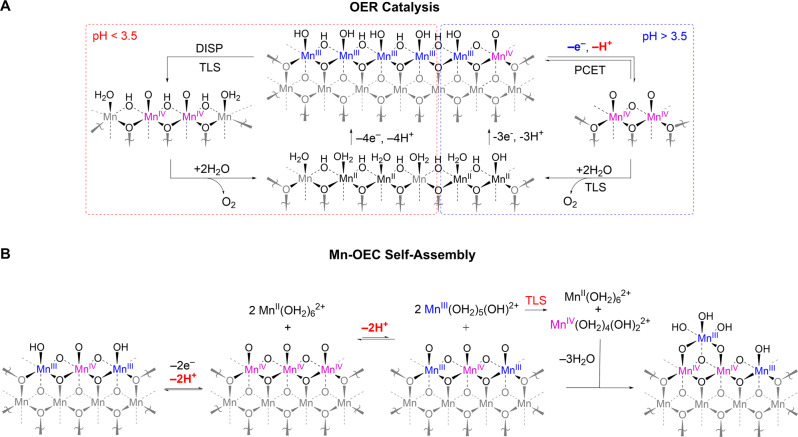
Mn-OEC catalysis and self-assembly. **A** Proposed OER catalytic cycle for Mn-OECs, as determined from spectroscopic analysis and electrochemical kinetics studies at different pHs. **B** Proposed mechanism for the generation of Mn-OEC films. The protons (H^+^) that appear in the electrochemical rate laws for OER and catalyst deposition/regeneration are highlighted in red. Owing to the disproportionation reaction involving two manganese complexes, an inverse fourth-order proton dependence is observed, enabling self-healing to be preserved at pH > –0.5. DISP and TLS denote disproportionation and turnover-limiting step, respectively.

Modulating the relationship between potential and pH for OER and self-assembly can lead to extraordinary catalytic properties. Self-healing in a Mn-OEC produced by electrodeposition from dilute Mn^2+^ solutions in a weakly basic electrolyte furnishes a stable OER catalyst at pHs as low as –0.5^73–75^. The rate of OER by Mn-OECs in P_i_ and MeP_i_ electrolytes is zeroth order in proton activity at pH < 3.5 and inverse first order in proton activity at pH > 3.5. Based on Tafel analysis, two parallel OER mechanisms shown in Fig. 4A have been proposed in these different pH regions: (1) a Mn^3+^ disproportionation process with zeroth-order dependence on proton activity that predominates at pH < 3.5 and (2) a 1e^–^/1H^+^ pathway that is dominant at pH > 3.5. This modest pH dependence for OER is juxtaposed to a significant pH dependence for deposition. An inverse fourth-order dependence on proton activity arises from a turnover-limiting disproportionation reaction, which also gives rise to a second-order dependence on Mn^2+^ concentration^73^. This steep pH dependence for catalyst deposition results in a potential–pH diagram with a crossing for OER and catalyst deposition/regeneration at pH –0.5 (Fig. 3B), allowing the Mn-OEC to operate in concentrated acid. The functional stability of Mn-OECs is supported by ^31^P NMR line width analysis when using MeP_i_ as an electrolyte^74^. The stability of Mn-OECs, however, is complicated by the formation of MnO_4_^−^ ions at high potentials, preventing operation at high current densities (and attendant higher potentials) for OER^76^. As such, the stability of Mn-OECs in acidic electrolytes is maintained only at low OER current densities (< 1 mA cm^–2^). Activation of Mn-OECs by potential cycling affords a substantial improvement in OER activity, resulting in two orders of magnitude increase in current density at pH 2.5^73^. This activity enhancement for Mn-OECs upon varying the synthesis protocol^73,77^ suggests that the generation of acid-stable Mn-OECs that show higher OER activity is possible.

## Self-healing Ni-OECs

**Fig. 5:**
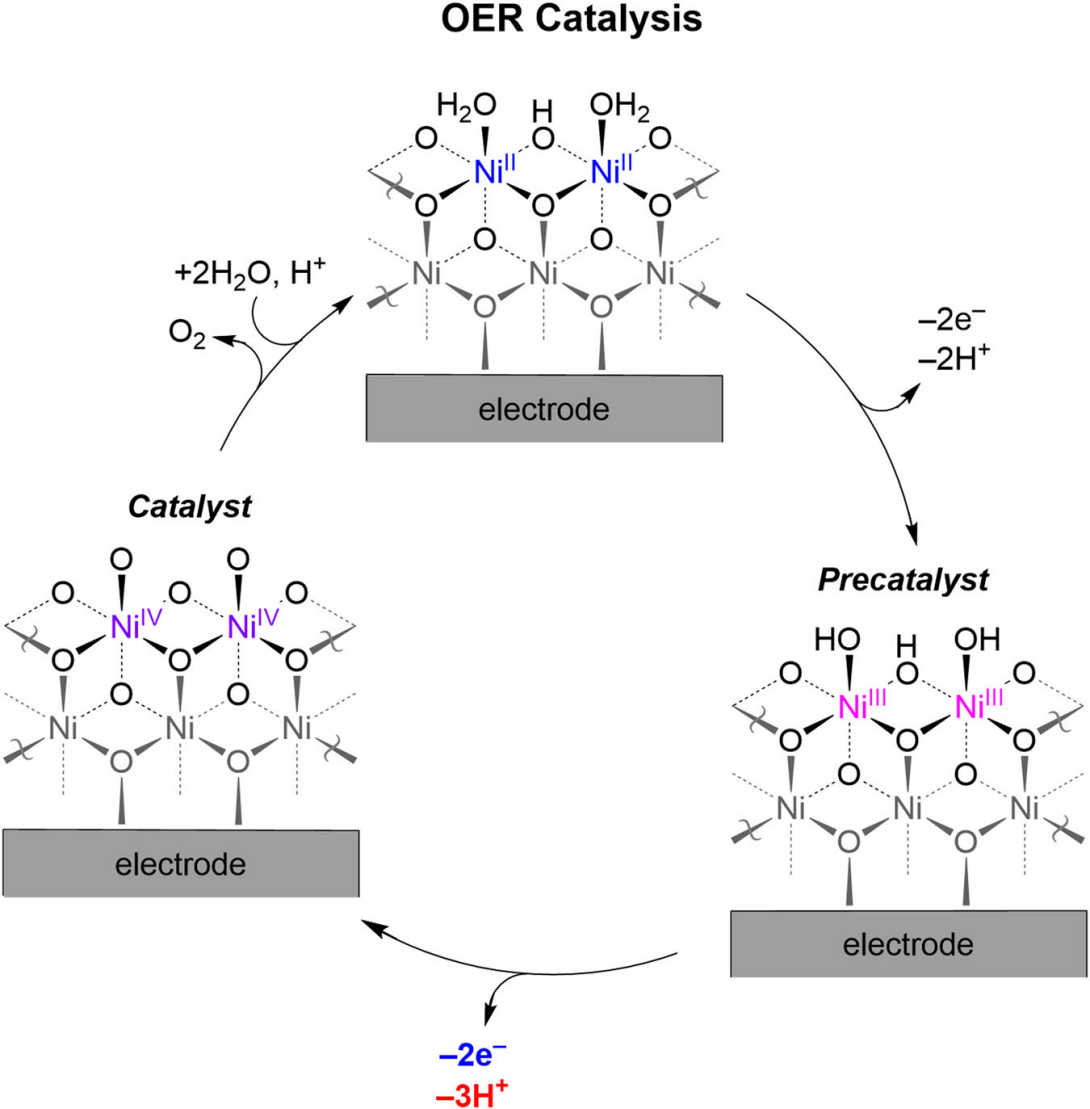
Ni-OEC catalytic cycle. Proposed OER catalytic cycle for Ni-OEC with B_i_ as an electrolyte, as determined from spectroscopic analysis and electrochemical kinetics studies for NiB_i_.

Self-healing Ni-OEC may be established with B_i_ as the electrolyte, as P_i_ is unable to support nickelate cluster formation^78–80^. The NiB_i_ catalyst is prepared by anodic electrodeposition from dilute aqueous Ni^2+^ solutions in the presence of B_i_ at pH 9.2. Subsequent activation by anodization affords enhanced OER activity owing to an average increase in the Ni oxidation state from +3.2 to +3.6, indicating substantial generation of formally Ni^4+^ species, whereas nonactivated films are predominately Ni^3+^^79,80^. The mechanism for NiB_i_ self-healing resembles that of CoP_i_ with the caveat that Tafel analysis of NiB_i_ furnishes an OER rate law that is inverse first order in [B_i_] (i.e., for 20–300 mM B_i_) and inverse third order in proton activity (i.e., for pH 8.5–12). The 2e^–^/3H^+^ pre-equilibrium followed by a rate-limiting O–O bond formation and O_2_ release is shown in Fig. 5. Despite the ostensible “inhibitory” effect of B_i_ on OER kinetics, the activity of NiB_i_ is high owing to the differences of B_i_ vs. P_i_ substitution. Whereas P_i_ must exchange at dicobalt edge sites by a dissociative substitution mechanism, B_i_ can exchange with more facility at edge sites via Lewis acid–base mechanism^56^:

The differences in the role of electrolyte between Co-OECs and Ni-OECs highlight the pivotal role that electrolytes play in OER and self-healing by facilitating proton transfer at intermediate pHs and by establishing the equilibrium for catalyst deposition/regeneration. The different characteristics of the PCET pre-equilibrium (and thus Tafel slope) for CoP_i_ and NiB_i_ and associated disparate pH dependences for these catalysts render NiB_i_ increasingly more active than CoP_i_ as the pH is elevated. However, CoP_i_ outperforms NiB_i_ at neutral and slightly acidic conditions^79^. On that note, Ni-OECs are not stable in acidic electrolytes at moderate overpotentials owing to dissolution of the oxides, as judged by the Pourbaix diagram of Ni in water^76^. In contrast, Ni-OECs are stable at alkaline pHs and display self-healing in concentrated base (e.g., 0.1–1.0 M KOH) devoid of buffer electrolytes^79^. Accordingly, Ni-OECs exhibit self-healing properties at pH ~9–14, however, the pH dependence of the Ni-OEC film assembly process remains to be defined to enable the construction of potential-pH diagrams such as those shown for Co-OECs and Mn-OECs in Fig. 3.

As with Co-OECs, catalytically active Ni-OEC films may form from molecular complexes that provide the requisite metal ions^81^. The use of a proton-accepting B_i_ electrolyte has been shown to be necessary to achieve and maintain high catalytic activity for these Ni-OECs at pH 9.2. The facility with which M-OEC films form from molecular complexes highlights the difficulties associated with deploying molecular systems as OER catalysts. Specifically, determining whether catalytic activity is derived from the initial molecular species or from the generation of small amounts of M-OECs, where the molecular complex serves as a metal source, remains a challenging task.

## Self-healing in Cu-OECs

Self-healing has been imparted to Cu-OECs where sustained OER is achieved in a carbonate buffer (pH ~ 10.8) containing sufficient dissolved Cu^2+^ to drive catalyst self-assembly^82^. Here, formation of a compact film of CuO on a Cu surface prevents anodic corrosion and enables sustained OER catalysis.

## Self-healing mixed-metal OECs

### Mixed-metal oxide OECs with catalytic and structural metals

**Fig. 6:**
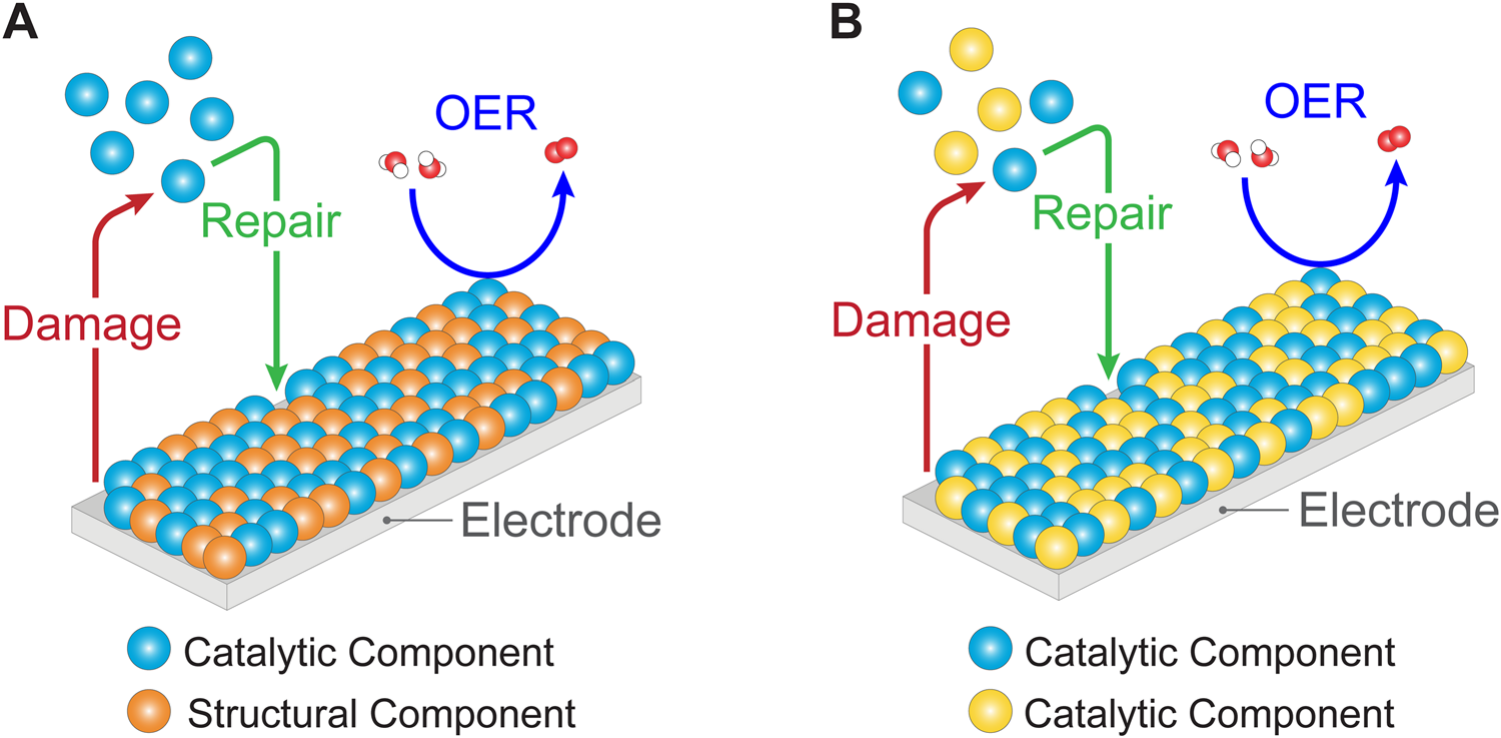
Schematic depiction of two common types of mixed-metal oxide OECs (M′M–OECs) that may possess self-healing properties. **A** Self-healing M′M–OEC system composed of separate catalytic (e.g., Co, Ni; blue spheres) and structural components (e.g., PbO_*x*_; orange spheres). **B** Self-healing M′M–OEC system comprising two types of catalytic components (blue, yellow spheres). Self-healing in both types of systems involves the catalytic component(s) undergoing damage and repair while performing OER. The colored spheres above the electrode surface denote dissolved catalyst capable of repair.

Self-healing may be augmented by alloying catalytically active metal elements with structural metal elements to further enhance stability. In a common class of mixed-metal oxide OECs (M′M–OECs), some components may themselves be formally self-healing while other components function predominantly as structural elements (Fig. 6A) such that OER activity and stability are decoupled and therefore may be optimized independently^83^. The incorporation of various catalytically active metals into a shared inert oxide framework can fortify the metal–oxygen bonds that are generally weakened during OER, particularly at active sites, leading to enhanced stability while preserving high OER activity. Among the most common types of M′M–OECs are systems where a catalytically competent metal (e.g., Mn, Fe, Co, Ni) is doped into a conductive and thermodynamically stable matrix (e.g., PbO_*x*_) to achieve materials with simultaneous high activity and stability under acidic conditions^83,84^. For example, the self-healing and structurally fortified CoFePbO_*x*_ is functionally stable under optimized reaction conditions (i.e., 5 mM Co^2+^, 1 mM Fe^3+^, 0.5 mM Pb^2+^) at both ambient and elevated temperatures in solutions of pH 0–1 with no loss of activity after even one week of continuous operation^85^. Since catalyst repair and damage, as well as OER, are all kinetically controlled processes, tuning the concentrations of the component metals, along with temperature and pH, appears to be a promising means of imparting self-healing to M′M–OECs.

### Mixed-metal oxide OECs with different catalytic metals

 Another common type of M′M–OECs is generated by alloying metals to increase the activity of a unary metal oxide that already displays OER activity (Fig. 6B). The introduction of Fe^3+^ into NiB_i_ and CoP_i_ has been shown to be especially effective at increasing OER activity^86–91^, though the reason for this enhancement continues to be explored^92^. As Fe-based OECs are not self-healing^93^, NiFe-OECs and CoFe-OECs incur a significant loss in stability owing to oxide dissolution under varying conditions^83,84^. Notwithstanding, a dynamically stable CoFe-OEC may be prepared using a nanorod array of CoMoO_4_ as a host matrix for the redeposition of FeOOH and CoOOH in alkaline media^94^. The resulting CoFe-OEC exhibits high OER activity, sustaining an OER current density of 100 mA cm^−2^ (based on geometric electrode surface area) with an overpotential of 298 mV in 1 M KOH. Furthermore, recent work has demonstrated that self-healing may also be achieved by operating in alkaline electrolytes containing a small amount of Fe^95,96^. For example, addition of 0.1 ppm Fe to the operating KOH electrolyte solution was shown to promote self-healing in a range of first-row transition metal oxyhydroxides by facilitating dynamic Fe exchange^95^. Isotopic labeling experiments with ^56^Fe and ^57^Fe revealed rapid exchange of isotopes while preserving overall Fe content, indicating a fast rate of Fe regeneration as compared to the rate of dissolution. Consequently, the electrolyte must contain a sufficiently high concentration of Fe so as to raise the rate of Fe regeneration to engender a high number of dynamic active sites, and thus preserve high OER activity and functional stability. In a related study, introduction of Co into NiFe-OECs was shown to promote redeposition of Fe in situ and, thus, engender catalyst self-healing when operating in strongly alkaline (pH 14) electrolytes containing both ferrous and borate ions^96^. Here, the Fe ions could only be redeposited on sites adjacent to Co sites, preventing the deposition of thick Fe oxyhydroxide overlayers. This unique self-limiting thickness was demonstrated to be ideal for integration with photoelectrodes as a high light transmittance through the catalyst layer could be maintained during OER operation. A similar approach has been taken with NiFe-OECs in carbonate buffer, where long-term stability and self-healing was induced by adding Ni^2+^ and Fe^3+^ to the operating electrolyte in weakly to strongly basic solutions^97^. Together these examples highlight that the competing rates of metal oxide repair and damage may be influenced by tuning the concentrations of dissolved component metals in solution.

### Other mixed-metal oxide OECs

Self-healing M′M-OECs beyond typical electrodeposited metal oxide films have also been reported. A NiFe-OEC generated in situ on a Mo-doped BiVO_4_/Ni/Sn photoelectrode is self-healing in a site-specific manner when operated in a B_i_ electrolyte by using a passivated Ni contact layer as the source of Ni^2+^^98^. Similarly, dynamic cycling of dissolution, diffusion, and deposition of Ni and Fe in a Si-based photoanode coated with a dual layer electrocatalyst engenders extended electrode stability^99^. Furthermore, a nanoparticle-based system composed of NiFe-layered double hydroxide nanoparticles deposited onto a Ni electrode has been shown to possess self-healing properties in an alkaline electrolyser^100^. This catalyst is generated in situ during electrolysis driven by electrostatic forces; self-healing is induced by operating in an electrolyte containing the component nanoparticles that continually deposit onto the underlying Ni electrode during operation. Along these lines, a ligand-induced self-healing mechanism was recently reported for a Fe-based electrocatalyst operating in strongly alkaline media^101^. These self-healing approaches are analogous to those described above but rely on a component material or a stabilizing ligand, as opposed to a dissolved metal ion, to direct the damage vs. repair equilibrium toward repair.

 Finally, self-healing may directly involve oxide lattice sites. For perovskite systems such as SrCoO_3_^102^, self-healing is derived from surface adsorbed H_2_O molecules, which dissociate to form reactive oxygen atoms that fill oxygen vacancies on the surface produced during OER. As the formation of such oxide vacancies define catalyst degradation, their depletion accommodates catalyst repair. This system is self-healing as the onset potential required to drive the filling of the oxygen vacancy is lower than the potential at which OER proceeds. A similar oxygen vacancy-based self-healing property has been proposed for TiO_2_^103^ and α-MnO_2_^104^ OECs.

## Summary and outlook

 The need to implement sustainable energy sources on a global scale has promoted H_2_ as a potential energy carrier. Electrochemical water splitting provides a scalable route to H_2_ production from renewable sources and has prompted the development of OECs to drive the more challenging oxygen evolution half-reaction. In this effort, self-healing OECs based on earth-abundant first-row transition metal oxides have garnered significant attention owing to their ability to drive OER at relatively low overpotentials while remaining stable under various operating conditions. Self-healing unary metal oxide OECs such as MnO_*x*_, CoP_i_, and NiB_i_ provide activity and stability in acidic, neutral, and basic conditions, whereas emerging mixed-metal oxide OECs such as CoFePbO_*x*_ and NiFeO_*x*_ offer improved activity and/or stability across a similar range of conditions. Under a wide range of operating conditions, OER catalysts may be more easily interfaced with (1) materials for direct conversion of water to oxygen and hydrogen at high efficiency^24–26,105^ and (2) biological organisms to perform artificial photosynthesis^8,9^. A better fundamental understanding of damage and repair mechanisms in existing and future systems may expand the self-healing capabilities of OECs to a wider range of conditions and promote self-healing as a general design principle in catalysis development, opening paths to a broad set of future applications. Overall, this review has highlighted the prospects for self-healing first-row transition metal oxides as OECs in water splitting systems. The increasing need to transition away from fossil fuels necessitates further research into self-healing OECs based on earth-abundant elements, expanding the practicality and utility of these versatile chemical platforms.
